# Differentiated Evolutionary Strategies of Genetic Diversification in Atlantic and Pacific Thaumarchaeal Populations

**DOI:** 10.1128/msystems.01477-21

**Published:** 2022-06-13

**Authors:** Yunha Hwang, Peter R. Girguis

**Affiliations:** a Department of Organismic and Evolutionary Biology, Harvard University, Cambridge, Massachusetts, USA; Scripps Institution of Oceanography

**Keywords:** microdiversity, metagenomics, time series analysis, population genetics, strain-level diversity, ammonia-oxidizing archaea, pangenome, comparative genomics, auxiliary genes, accessory genes, phosphorus limitation, comparative studies

## Abstract

Some marine microbes are seemingly “ubiquitous,” thriving across a wide range of environmental conditions. While the increased depth in metagenomic sequencing has led to a growing body of research on within-population heterogeneity in environmental microbial populations, there have been fewer systematic comparisons and characterizations of population-level genetic diversity over broader expanses of time and space. Here, we investigated the factors that govern the diversification of ubiquitous microbial taxa found within and between ocean basins. Specifically, we use mapped metagenomic paired reads to examine the genetic diversity of ammonia-oxidizing archaeal (“*Candidatus* Nitrosopelagicus brevis”) populations in the Pacific (Hawaii Ocean Time-series [HOT]) and Atlantic (Bermuda Atlantic Time Series [BATS]) Oceans sampled over 2 years. We observed higher nucleotide diversity in “*Ca.* N. brevis” at HOT, driven by a higher rate of homologous recombination. In contrast, “*Ca.* N. brevis” at BATS featured a more open pangenome with a larger set of genes that were specific to BATS, suggesting a history of dynamic gene gain and loss events. Furthermore, we identified highly differentiated genes that were regulatory in function, some of which exhibited evidence of recent selective sweeps. These findings indicate that different modes of genetic diversification likely incur specific adaptive advantages depending on the selective pressures that they are under. Within-population diversity generated by the environment-specific strategies of genetic diversification is likely key to the ecological success of “*Ca.* N. brevis.”

**IMPORTANCE** Ammonia-oxidizing archaea (AOA) are one of the most abundant chemolithoautotrophic microbes in the marine water column and are major contributors to global carbon and nitrogen cycling. Despite their ecological importance and geographical pervasiveness, there have been limited systematic comparisons and characterizations of their population-level genetic diversity over time and space. Here, we use metagenomic time series from two ocean observatories to address the fundamental questions of how abiotic and biotic factors shape the population-level genetic diversity and how natural microbial populations adapt across diverse habitats. We show that the marine AOA “*Candidatus* Nitrosopelagicus brevis” in different ocean basins exhibits distinct modes of genetic diversification in response to their selective regimes shaped by nutrient availability and patterns of environmental fluctuations. Our findings specific to “*Ca.* N. brevis” have broader implications, particularly in understanding the population-level responses to the changing climate and predicting its impact on biogeochemical cycles.

## INTRODUCTION

Marine microbial populations are shaped by a complex interplay of dispersal, drift, and selection (1). While currents connect global oceans, there exists heterogeneity in the nutrient availability and environmental variables across the marine water column, forming distinct ecosystems with differential selective pressures across spatial scales (2). Previous global surveys (3, 4) of the marine water column microbiome revealed both ubiquitous and site-specific microbial populations that inhabit geographically distant waters. In particular, much research has been done on the globally widespread and abundant marine microbial taxa (e.g., *Prochlorococcus* [5] and SAR11 [6]), also referred to as “keystone taxa” (7), that often occupy important functional niches in the marine ecosystem. With increased resolution in both sampling and genome sequencing, there is growing evidence that marine microbial populations with massive population sizes consist of highly diverse strains and subpopulations with flexible genomes, representing populations with high within-population diversity (8–11). Therefore, there is an increasing research interest in understanding how these ubiquitous microbial taxa adapt to different selective regimes in diverse and dynamic ocean environments and how microbial adaptation and natural selection are encoded in their genomic diversity.

Ammonia-oxidizing archaea (AOA) belonging to the phylum “*Candidatus* Thaumarchaeota” (or the GTDB phylum *Thermoproteota* and class *Nitrososphaeria*, according to the standardization of archaeal taxonomy proposed recently by Rinke et al. [12]) are another example of ubiquitous and abundant microbial taxa that play an important role in the carbon and nitrogen turnover of the marine ecosystem. AOA fix carbon and occupy a functional niche by generating energy through nitrification. AOA are particularly abundant in the upper ocean (13, 14) as well as the mesopelagic zones (15) and have been shown to dominate the nitrification process in the global oceans (16, 17). “*Candidatus* Nitrosopelagicus brevis” (18) is an AOA species found to be abundant across the lower euphotic zone in the oligotrophic ocean (19) and characterized by a highly streamlined genome of ~1.2 Mbp. Despite its small genome, “*Ca.* N. brevis” is one of the most ecologically successful archaeal species in the ocean, being found across wide geochemical and environmental gradients, with evidence of strain-level diversity resulting in physiological differentiation in nitrogen source (e.g., urea) utilization (20, 21). Previous studies of AOA abundance and function in the upper ocean have revealed significant seasonality, with decreased abundance and activity in summers (22–25), indicating population-level responses to environmental fluctuations. Due to their ecological importance, there has been increasing research interest in temporal variations in the population structure and adaptive strategies of AOA populations in different environments (21, 26–29). However, there have been limited systematic surveys of the within-population diversity of AOA, and we know little about the mechanisms of diversity generation and maintenance in natural AOA populations.

In order to understand how the within-population diversity of AOA varies over space and time, we compared “*Ca.* N. brevis” populations over 2 years between two well-studied sites, Hawaii Ocean Time-series (HOT) station ALOHA (30) in the Pacific Ocean and the Bermuda Atlantic Time Series (BATS) station (31) in the Atlantic Ocean. These sites are comparable in that they are both oligotrophic with similar levels of primary production (8). However, there are key ecological distinctions between the two sites in both nutrient availability and seasonal variations in water column stratification. For example, BATS experiences stronger seasonal fluctuations in light, temperature, and nutrient concentrations than HOT, with deeper mixing (~150 to 200 m) in winters (31, 32). Additionally, they differ in their geochemistry, with notably lower inorganic phosphorus concentrations at BATS than at HOT (33–35) and higher inputs of iron and other metals at HOT than at BATS (36). Previous surveys of the rates of nitrification and ammonia oxidation in these two sites revealed high degrees of temporal variability (37, 38), suggesting that AOA populations in these locations may experience dynamicity over time.

To better understand how an ecologically “successful” species of microbe adapts across diverse environmental gradients, we compared the “*Ca.* N. brevis” populations of HOT and BATS to address the following questions. (i) Do “*Ca.* N. brevis” populations differ in their levels of nucleotide diversity in different selective regimes? (ii) Do “*Ca.* N. brevis” gene contents differ between the ocean basins? (iii) Are “*Ca.* N. brevis” populations shaped by genome-wide or gene-specific selective sweeps? We first investigated the genetic diversity of “*Ca.* N. brevis” populations in metagenomes from the HOT and BATS sites over a 2-year time series and contextualized our findings by comparing them with those for other abundant community members in the ecosystem. We then used mapped metagenomic reads to calculate the nucleotide diversity and identify single nucleotide variants (SNVs) and their linkage in each sample. We conducted pangenomic analyses to identify basin-specific genes in “*Ca.* N. brevis” populations and then analyzed the shared fraction of the genome to determine highly differentiated alleles between the two sites. SNV profiles were compared over time to delineate temporal fluctuations in population structure and their correlation with environmental variables.

## RESULTS

### “*Ca.* N. brevis” is abundant at both HOT and BATS but more diverse at HOT.

**FIG 1:**
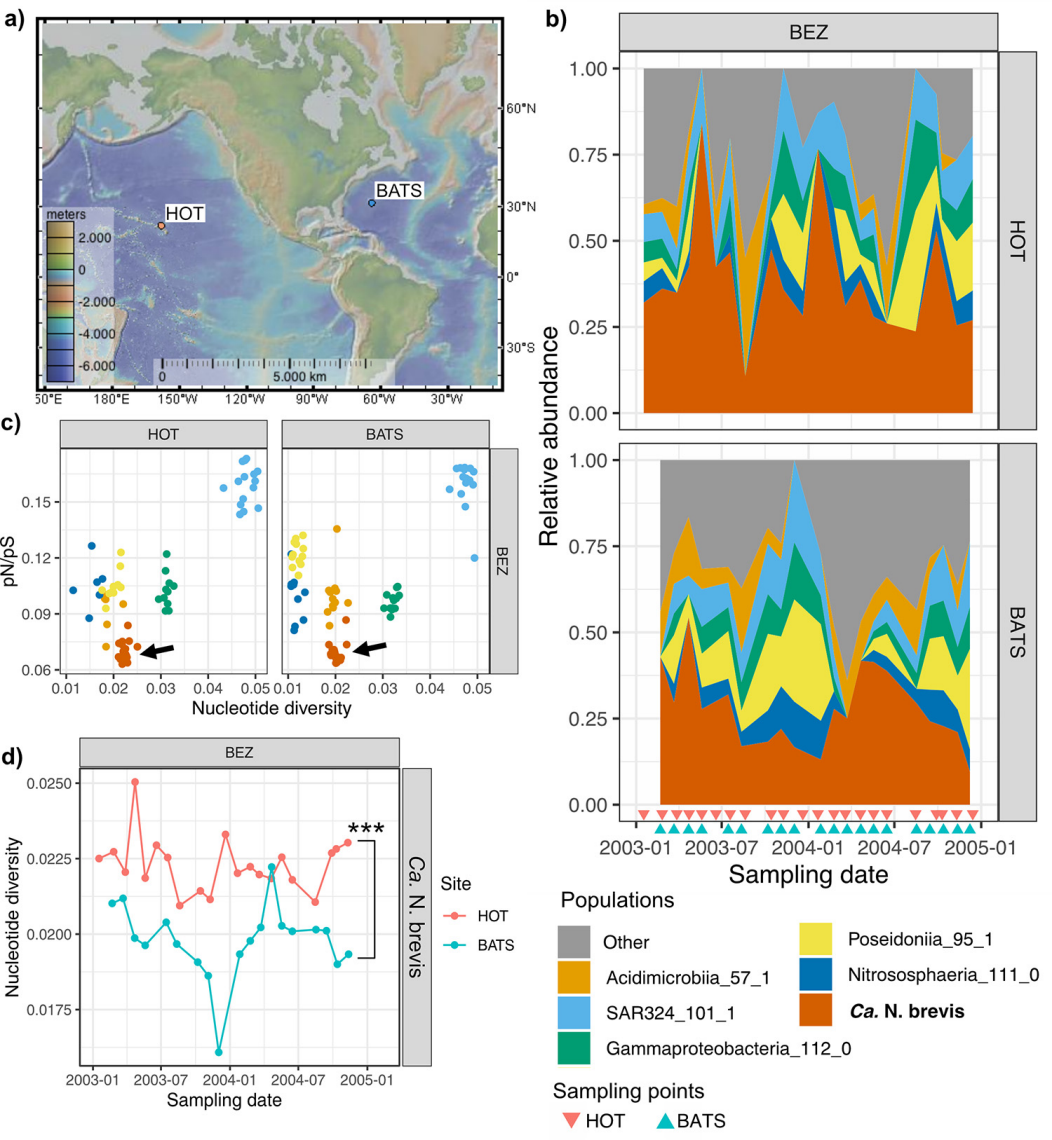
Microbial populations in the below-euphotic zones (BEZ) of the Hawaii ALOHA Time-series (HOT) and the Bermuda Atlantic Time Series (BATS). (a) Sampling locations. (b) Microbiome structures of the BEZ samples in both sites over an ~2-year sampling period. The top five most abundant species are highlighted. Sampling points are labeled with color-coded triangles along the *x* axis. (c) *pN*/*pS* ratios and nucleotide diversity of the top six most abundant species. Each point on the plot depicts a species-level population in a sample, detected with at least 10× coverage and 0.8 breadth. Data points are color-coded using the same designation as the ones in panel b. “*Ca.* N. brevis” population clusters are highlighted with arrows. (d) Change in the nucleotide diversity of “*Ca.* N. brevis” populations in BEZ samples at HOT and BATS over the sampling period. Statistically significant differences between the two sites are noted with asterisks (*P* < 1E−6 [by a paired *t* test]).

Across 130 metagenomes from HOT and BATS (see Fig. 1a for sampling locations; see also Table S1 in the supplemental material for metagenome metadata), we binned 891 above-medium-quality (>70% completeness and <10% contamination) (39) metagenome-assembled genomes (MAGs), which were dereplicated into 170 representative MAGs (rMAGs). Of these rMAGs, 76 were present (>5× coverage and >0.5 breadth using mapped reads) at both sites, 61 were specific to BATS, 31 were specific to HOT, and 2 could not be detected in either sample with sufficient abundance and confidence (for further details on the rMAGs, see Table S2). The community composition and structure stayed stable over time, with statistically significant seasonal fluctuations being observed in the surface water (SW) samples at BATS (*P* = 0.001 [by permutational multivariate analysis of variance {PERMANOVA}]) (for the changes in the community profile over time, see Fig. S1 at https://doi.org/10.6084/m9.figshare.19357958). An rMAG belonging to the “*Ca.* Nitrosopelagicus brevis” species (rMAG_Nbrevis) was the second most abundant rMAG (after *Prochlorococcus*) across all samples and the most abundant in the samples below the euphotic zone (BEZ samples) at both sites (Fig. 1b; see also Fig. S2 at https://doi.org/10.6084/m9.figshare.19357964 for the relative abundances of the top six most abundant rMAGs across samples), with relative abundances ranging between 5.7 and 84.1%. rMAG_Nbrevis was 1.13 Mbp, smaller than the reference genome of strain CN25 (1.23 Mbp) and missing the two putative genomic islands identified previously (19). We predicted 1,408 genes in rMAG_Nbrevis and estimated it to be 99.27% complete, with no contamination. Interestingly, we found “*Ca.* N. brevis” MAGs to feature high codon usage bias (CUB), and we estimated “*Ca.* N. brevis” to have the shortest minimal doubling time (3.5 ± 2.2 h) (*n* = 79 medium- and high-quality “*Ca.* N. brevis” MAGs assembled across all sampling depths) out of the eight most frequently detected populations (see Fig. S3 at https://doi.org/10.6084/m9.figshare.19357982). This is surprising and inconsistent with the findings from previous culture studies of “*Ca.* N. brevis” C25 and U25, with reported doubling times of 7 days (20), which are significantly longer than the observed doubling time of 1 day for *Prochlorococcus* (40). It is possible that the *in situ* doubling time of the “*Ca.* N. brevis” population is much shorter and/or that there are other evolutionary processes at play that led to the observed particularly prominent CUB in “*Ca.* N. brevis” genomes. Notably, we detected three other much less abundant thaumarchaeal rMAGs: an AOA belonging to an unidentified species of the “*Ca.* Nitrosopelagicus” genus and two non-ammonia-oxidizing heterotrophic *Thaumarchaeota* (UBA57) members (41) (see Fig. S4 at https://doi.org/10.6084/m9.figshare.19357970). Our subsequent analyses focused on the populations of rMAG_Nbrevis in the BEZ samples because of their high abundance (read mapping coverage of >5×, the minimum threshold to reliably detect minor alleles [42]) throughout the 2-year sampling period.

10.1128/msystems.01477-21.3 TABLE S1 Sample metadata. All samples used in this study and the metadata (date, sampling depth, temperature [degrees Celsius], salinity [parts per trillion], library size, NCBI SRA accession numbers for reads and contigs, and assembly methods) are shown. Download Table S1, CSV file, 0.03 MB. Copyright © 2022 Hwang and Girguis. 2022 Hwang and Girguis. https://creativecommons.org/licenses/by/4.0/ This content is distributed under the terms of the Creative Commons Attribution 4.0 International license.

10.1128/msystems.01477-21.4 TABLE S2 rMAG metadata. Quality and statistics (contamination, completeness, strain heterogeneity, size, *N*_50_, broad taxonomic assignments, and ocean specificity) of dereplicated rMAGs that were used to delineate genetically defined populations in this study are shown. Download Table S2, CSV file, 0.03 MB. Copyright © 2022 Hwang and Girguis. 2022 Hwang and Girguis. https://creativecommons.org/licenses/by/4.0/ This content is distributed under the terms of the Creative Commons Attribution 4.0 International license.

We detected polymorphisms (or single nucleotide variants [SNVs]) in 5.28% ± 1.1% of the sites across rMAG_Nbrevis. SNVs were spread evenly across the genome (see Fig. S5 at https://doi.org/10.6084/m9.figshare.19357991): 74.8% ± 1.5% of the SNVs were synonymous, 15.8% ± 0.9% were nonsynonymous, and 5.0% ± 0.3% were intergenic. Such a low ratio of nonsynonymous to synonymous SNVs (*pN*/*pS* ratio) (0.068 [±0.007]) signifies that these populations have undergone purifying selection (43). We calculated the nucleotide diversity and *pN*/*pS* ratios of all rMAGs across samples and found that “*Ca.* N. brevis” populations have the lowest *pN*/*pS* ratio (0.068 ± 0.007) at both sites and a medium level of nucleotide diversity (π, 0.020 ± 0.002) (Fig. 1c visualizes the five most abundant species). We estimated the lower bound of the effective population size (*N_e_*) for rMAG_Nbrevis to be ~2.3 × 10^9^ based on the nucleotide diversity (π_neutral_ = 0.284 ± 0.037) of nonconserved third codon positions (*n* = 25,839 ± 5,296). This is within the same order of magnitude as what has been described (*N_e_* = ~1.5 × 10^9^) for the prochlorococcal ecotype using single-cell genomes (9) and similar to the estimation (*N_e_* = ~2.6 × 10^9^) for the most abundant *Prochlorococcus* rMAG in our data set (Cyanobacteriia_123_1).

“*Ca.* N. brevis” populations at HOT had statistically higher nucleotide diversity than those at BATS (*P* < 1E−6 [by a paired *t* test]; *t* = 7.5). In addition, we observed only minor fluctuations in nucleotide diversity in “*Ca.* N. brevis” populations, with no statistically significant difference in variance between the sites (*P* > 0.05 [by an F test]) over the 2-year sampling period (Fig. 1d). As established previously (42), nucleotide diversity was not correlated with coverage in these samples (see Fig. S6 at https://doi.org/10.6084/m9.figshare.19358003). Statistically significant differences (*P* < 0.05 [by Welch’s *t* test]) in nucleotide diversity between the sites were observed in 35 out of 75 non-“*Ca.* N. brevis” rMAGs that were present at both sites. The majority (31 out of 35) of the populations were more diverse at HOT than at BATS.

### “*Ca.* N. brevis” within-population diversity is maintained by a high recombination-to-mutation ratio.

**FIG 2:**
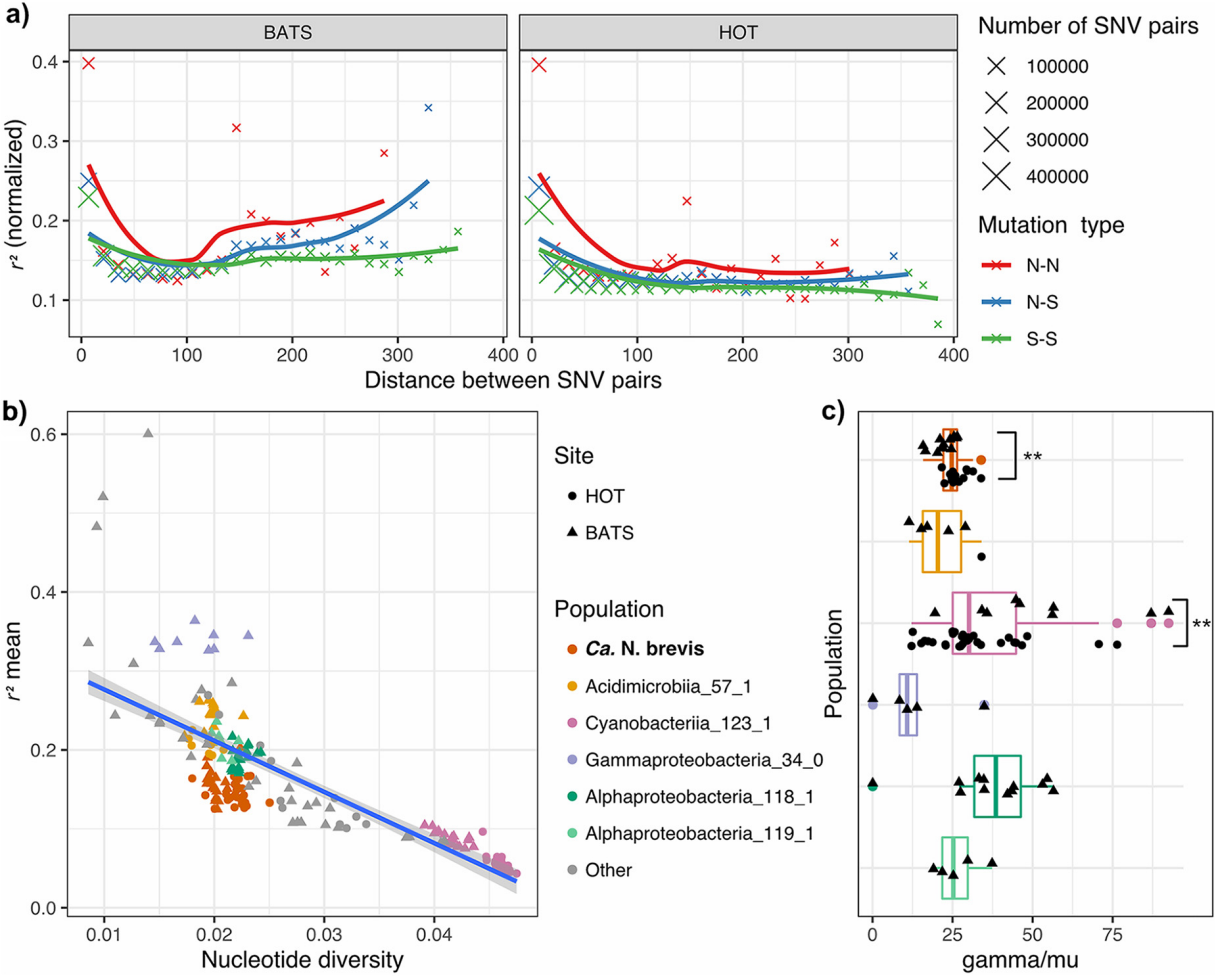
Recombining populations of “*Ca.* N. brevis.” (a) Decreasing normalized *r*^2^ metric with increasing distance between pairs of SNVs in protein-coding sequences. Pairs of SNVs were categorized based on the resulting amino acid change, where N-N denotes a pair of nonsynonymous SNVs, N-S denotes a pair of synonymous and nonsynonymous SNVs, and S-S denotes a pair of synonymous SNVs. The number of SNV pairs included to calculate the average *r*^2^ metric in a distance interval is visualized by the size of the data point. (b) Populations across the entire data set (SW, BEZ, and DCM) with >30× coverage displaying a diverse range of linkages between SNVs. Each point on the plot depicts a species-level population in a sample, and the shape of the point denotes the site from which it was derived. The top five most frequently observed species-level populations are color-coded. (c) Recombination-to-mutation ratio (gamma/mu) calculated for the top five most abundant populations with 50× coverage. Statistically significant differences between the gamma/mu values of the two sites are noted with asterisks (*P* < 0.05 [by Welch’s *t* test]).

Homologous recombination plays an important role in generating and maintaining the genetic diversity of microbial populations. In order to estimate the rate of recombination, we first examined the linkage of SNVs across the genome. Using the paired-end reads (2× 150 bp) with a median insert size of ~250 bp, we calculated the linkage of SNV pairs that were found up to 420 bp apart. A decay in linkage disequilibrium, which is characteristic of recombination, was observed in the “*Ca.* N. brevis” populations at both sites, as signified by the decrease in the *r*^2^ metric of linkage with increasing distance between SNVs (Fig. 2a). We also observed more linkage between nonsynonymous SNVs (N-N linkage) than between synonymous SNVs (S-S) or between nonsynonymous and synonymous SNVs (N-S) (Fig. 2a). This pattern of linkage can result from selective sweeps of nonsynonymous alleles and has previously been identified in genetically diverse populations of soil bacteria (44), in populations of Neisseria gonorrhoeae undergoing balancing selection and adaptive horizontal gene transfer (HGT) (45), and in phylogroup-specific genes of *Listeria* species (46). The average normalized *r*^2^ values across the genome (mean *r*^2^) for each population varied significantly between different taxa and were highly negatively correlated with nucleotide diversity (Spearman’s rho = −0.87; *P* < 1E−10) (Fig. 2b). We estimated the ratio of the recombination rate to the mutation rate (gamma/mu) of “*Ca.* N. brevis” to be 24.6 (±4.1), approaching levels similar to those of previously described quasisexual microbial populations of *Prochlorococcus* (47), *Synechococcus* (48), and *Vibrio* (49, 50). We estimated the recombination rates of the four other most abundant populations at SW and deep chlorophyll maximum (DCM) sampling depths (Fig. 2c) and found gamma/mu values similar to those of “*Ca.* N. brevis.” Interestingly, for populations present at both sites (“*Ca.* N. brevis” and Cyanobacteriia_123_1 [*Prochlorococcus* spp.]), we detected statistically significant differences in gamma/mu values (*P* < 0.05 [by Welch’s *t* test]) between the sites (Fig. 2c), with higher recombination rates estimated for the “*Ca.* N. brevis” populations at HOT (26.7 ± 3.45) than for those at BATS (22.2 ± 3.48) and higher recombination rates for the *Prochlorococcus* populations at BATS (52.5 ± 24.1) than for those at HOT (31.8 ± 15.0). This suggests the possibility of site-specific environmental influences on the recombination rate of populations of the same species.

### “*Ca.* N. brevis” at BATS has a larger pangenome with basin-specific accessory genes.

**FIG 3:**
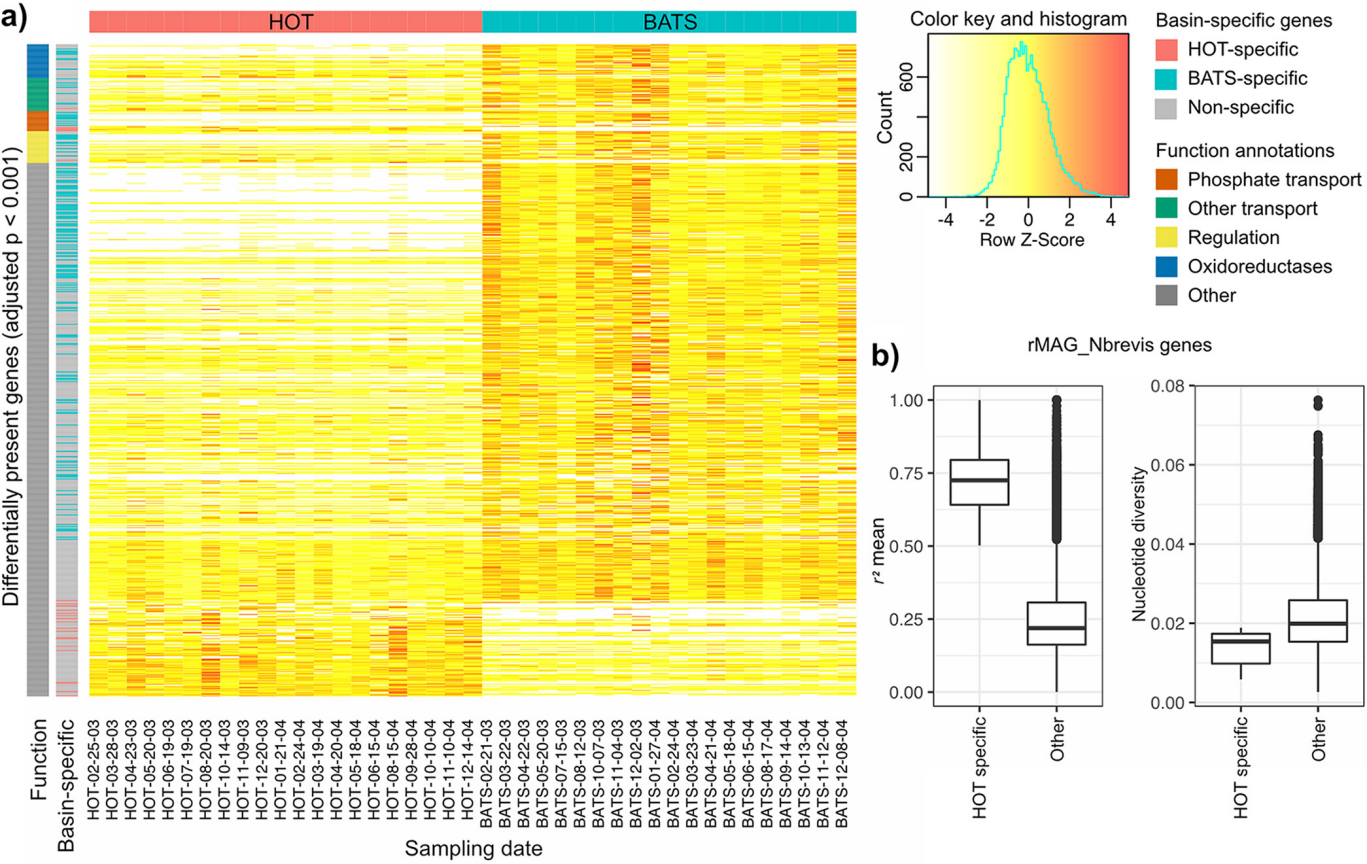
Larger pangenome of “*Ca.* N. brevis” populations at BATS. (a) Differentially present genes with statistical significance (adjusted *P* value of <0.001 [by ANOVA]) and their abundances relative to the “*Ca.* N. brevis” populations (estimated by the genome coverage) over time. The first row-side colors denote frequently observed functions, and the second row-side colors highlight basin-specific genes, defined as those not present in the other ocean at >10% of the expected abundance in any of the samples across the 2-year period. (b) Basin-specific genes in rMAG_Nbrevis with elevated linkage (*r*^2^) values and lower nucleotide diversity. rMAG_Nbrevis, which was binned from a HOT sample, contained two HOT-specific genes, encoding a phosphorus transporter and a transport regulator.

We observed a high variation in gene content among the “*Ca.* N. brevis” MAGs across samples. We compared the pangenome openness with those of three other abundant bacteria found in the same data set (*Prochlorococcus*, SAR11, and SAR324) (see Fig. S7 at https://doi.org/10.6084/m9.figshare.19358024) and found that “*Ca.* N. brevis” featured a level of pangenome openness similar to those of *Prochlorococcus* and SAR324, while SAR11 exhibited a much more closed pangenome. Using the 43 “*Ca.* N. brevis” MAGs binned (>98% average nucleotide identity [ANI] with each other), we evaluated the “*Ca.* N. brevis” pangenome across HOT and BATS and examined the differential coverage of genes between the BEZ samples of the two sites. The pangenome consisted of 3,316 genes, with 3,099 of these being sufficiently long for read mapping. We removed 258 genes that were mapped at a coverage higher than three times the average coverage of rMAG_Nbrevis in the corresponding sample, as they likely represented genes shared with other species or binning errors. The coverage of pangenome genes relative to the average genome coverage varied significantly among genes in a sample, indicating within-population variation in gene content across multiple strains (see Fig. S8 at https://doi.org/10.6084/m9.figshare.19358027). In addition, we found distinct gene contents between sites; we identified 616 genes that were differentially abundant between the sites (51) (adjusted *P* value of <0.001 by analysis of variance [ANOVA]) (Fig. 3a). Of the differentially abundant genes, we further identified 182 genes that were basin specific, defined as detected (coverage relative to the average genome coverage of >10%) at one site but not detected in any sample from the other site. Interestingly, 158 of the basin-specific genes were specific to BATS, indicating a larger pangenome of “*Ca.* N. brevis” populations at BATS (see Fig. 5a). Levels of pangenome openness were compared between the sites using 22 “*Ca.* N. brevis” MAGs from HOT BEZ and 21 from BATS BEZ, and we estimated “*Ca.* N. brevis” to have a more open pangenome by approximately 2-fold in BATS BEZ (γ = 0.44) than in HOT BEZ (γ = 0.18) (see Fig. S9 at https://doi.org/10.6084/m9.figshare.19358030). In order to confirm that this finding is likely not an artifact of the higher completeness of “*Ca.* N. brevis” MAGs binned from BATS, we compared the completenesses of the 43 BEZ “*Ca.* N. brevis” MAGs included in the pangenome analysis and found that those binned from HOT featured statistically higher completeness (*P* < 0.01 [by Welch’s *t* test]) (see Fig. S10 at https://doi.org/10.6084/m9.figshare.19358033), suggesting that the difference in the actual pangenome sizes between the two sites may be even larger than that observed in this metagenomic analysis.

**FIG 4:**
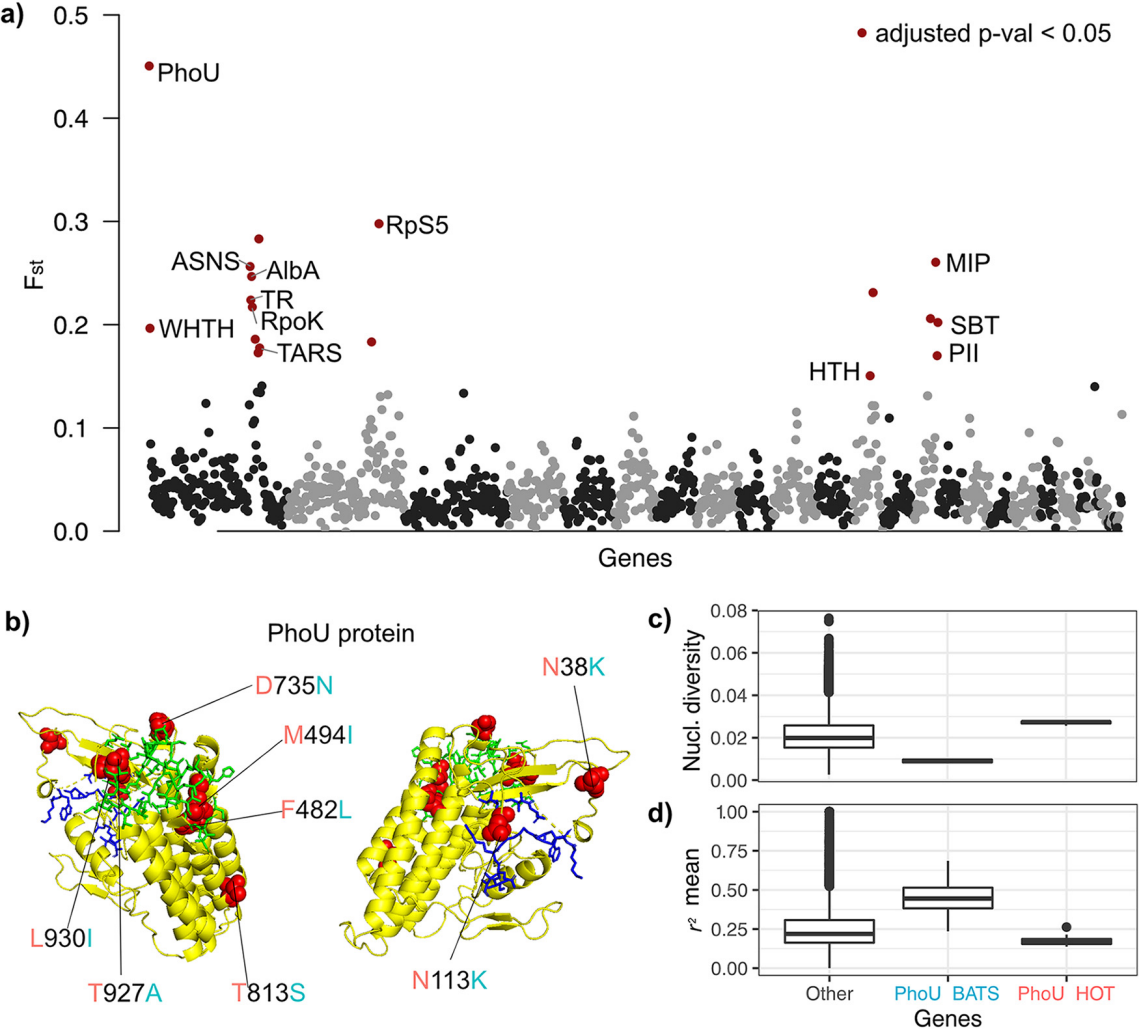
Differentiated alleles in the shared gene set between HOT and BATS “*Ca.* N. brevis” populations. (a) Differentiated genes (marked in red) with elevated *F_ST_* values with statistical significance (adjusted *P* value of <0.05 [by a Z test]). Abbreviations for putative gene functions are as follows: PhoU, phosphate-specific transport system accessory protein; TR, putative transcription regulator; ASNS, asparagine synthase; AlbA, DNA/RNA-binding protein AlbA; TARS, threonine-tRNA ligase; WHTH, winged-helix-turn-helix DNA-binding protein; MIP, MIP channel protein; RpS5, 30S ribosomal protein S5; RpL27, 50S ribosomal protein L27; HTH, HTH_45 domain-containing protein; PII, putative nitrogen regulatory protein P-II. (b) Predicted structure of the PhoU protein and nucleotide substitutions between HOT (pink) and BATS (light blue). Two putative pockets are depicted in dark blue and green rods. Substitutions are highlighted with red spheres, and the corresponding amino acid changes are labeled. (c) *F_ST_* values between all pairs of HOT and BATS populations of the top three most abundant populations in the BEZ besides “*Ca.* N. brevis.” (d) Elevated linkage (normalized mean *r*^2^) for highly differentiated genes (statistically high *F_ST_*) (adjusted *P* value of <0.05).

We examined the putative functions of the differentially present accessory genes and found 19 genes that were involved in phosphate transport. Interestingly, the high-affinity and low-velocity phosphate transporter complex PstSABC (52) (see Fig. S11a at https://doi.org/10.6084/m9.figshare.19358042) was approximately 7-fold more abundant at BATS, while the phosphonate transport system PhnCDE (53) was specific to BATS (see Fig. S11b at https://doi.org/10.6084/m9.figshare.19358042). In contrast, the low-affinity and high-velocity inorganic phosphate transport (Pit) (52) system was specific to HOT (see Fig. S11a at https://doi.org/10.6084/m9.figshare.19358042). Interestingly, the two phosphate systems (PstSABC and Pit) of differing affinity levels were found to substitute for each other at the same location in the genome, adjacent to a *phoU* gene (see Fig. S11a at https://doi.org/10.6084/m9.figshare.19358042). Furthermore, we identified phylogenetic diversity in the Pit system (a phosphate transport regulator [DUF47] family protein and *pit*) (see Fig. S12 at https://doi.org/10.6084/m9.figshare.19358048) detected in “*Ca.* N. brevis” MAGs assembled from HOT. This indicates that this locus (see Fig. S11a at https://doi.org/10.6084/m9.figshare.19358042) may be undergoing frequent substitutions of gene cassettes involved in phosphate transport. The diversity of phosphate transport systems was previously identified by Qin et al. (21); however, the basin-specific preference of the systems had not been identified to date. Basin-specific genes were functionally enriched in membrane transport, redox balance, and transcriptional regulation (Fig. 4a; see also Text S1 in the supplemental material for further details). rMAG_Nbrevis, derived from a HOT sample, contained two genes (DUF47 and *pit*) specific to HOT, for which we could calculate the *r*^2^ and nucleotide diversity values in the context of the rest of the genome. We found significantly higher linkage and lower nucleotide diversity values for these two genes than for the rest of the genome, suggesting the possibility of a recent selective sweep and/or a high level of purifying selection acting on these loci (Fig. 3b).

10.1128/msystems.01477-21.1 TEXT S1 Supplemental results on ocean-specific genes. Download Text S1, DOCX file, 0.01 MB. Copyright © 2022 Hwang and Girguis. 2022 Hwang and Girguis. https://creativecommons.org/licenses/by/4.0/ This content is distributed under the terms of the Creative Commons Attribution 4.0 International license.

### Regulatory genes are key to the differentiation of “*Ca.* N. brevis” populations at HOT and BATS.

In order to understand how “*Ca.* N. brevis” populations differ between HOT and BATS in the shared gene content, we calculated the fixation indices (*F_ST_*) of each shared gene in rMAG_Nbrevis between the two sites. The average *F_ST_* values across all shared genes between the HOT and BATS populations were relatively low, at 3.8% ± 3.2%, indicating that the alleles of most genes were dispersed between the two populations. However, we identified 14 genes with statistically high *F_ST_* values ranging up to 44.2% (adjusted *P* value of <0.05 [by a right-tailed test]) (Fig. 4a). Notably, these genes with high *F_ST_* values were found colocalized, possibly due to genetic hitchhiking in selective sweep events. We were able to predict the functions of 12 out of 18 genes with high *F_ST_* values and found that the majority of these genes have functions associated with transcriptional regulation. For instance, the gene with the highest *F_ST_* was one of the three *phoU* genes in rMAG_Nbrevis. Notably, this *phoU* gene was found adjacent to the Pst and Pit systems (see Fig. S11a at https://doi.org/10.6084/m9.figshare.19358042). We identified eight nonsynonymous substitutions in this *phoU* sequence at BATS and observed that these substitutions were enriched in or near the two putative pockets (Fig. 4b). Other high-*F_ST_* genes involved in transcription regulation were DNA/RNA-binding protein AlbA (AlbA), a winged-helix-turn-helix DNA-binding protein (WHTH), a putative nitrogen regulatory protein (PII), an HTH_45 domain-containing protein (HTH), and a putative transcription regulator (TR). Additionally, another large fraction of the high-*F_ST_* genes consisted of housekeeping genes, such as 30S ribosomal protein S5 (RpS5), DNA-directed RNA polymerase subunit K (RpoK), asparagine synthetase (ASNS), and threonine-tRNA ligase (TARS), and genes involved in transport, such as MIP channel protein (MIP) and sodium-dependent bicarbonate transport domain-containing protein (SBT). The *phoU* gene at BATS had significantly lower nucleotide diversity and higher mean *r*^2^ values, suggesting higher selective pressure on *phoU* at BATS than at HOT (Fig. 4c). Similar patterns in the mean *r*^2^ and nucleotide diversity characteristic of selection at BATS were observed for WHTH and MIP (see Fig. S13 at https://doi.org/10.6084/m9.figshare.19358054), both of which were located near *phoU* (see Fig. S11a at https://doi.org/10.6084/m9.figshare.19358042). Using consensus SNVs in neutral third positions in the codon, we estimated that “*Ca.* N. brevis” populations at the two sites diverged at least hundreds of thousands of years ago (see Text S2 in the supplemental material and Fig. S14 at https://doi.org/10.6084/m9.figshare.19358066 for more details).

10.1128/msystems.01477-21.2 TEXT S2 Supplemental results on the time of divergence between HOT and BATS populations. Download Text S2, DOCX file, 0.01 MB. Copyright © 2022 Hwang and Girguis. 2022 Hwang and Girguis. https://creativecommons.org/licenses/by/4.0/ This content is distributed under the terms of the Creative Commons Attribution 4.0 International license.

### HOT “*Ca.* N. brevis” populations exhibit fluctuations in population structure that correlate with temperature.

**FIG 5:**
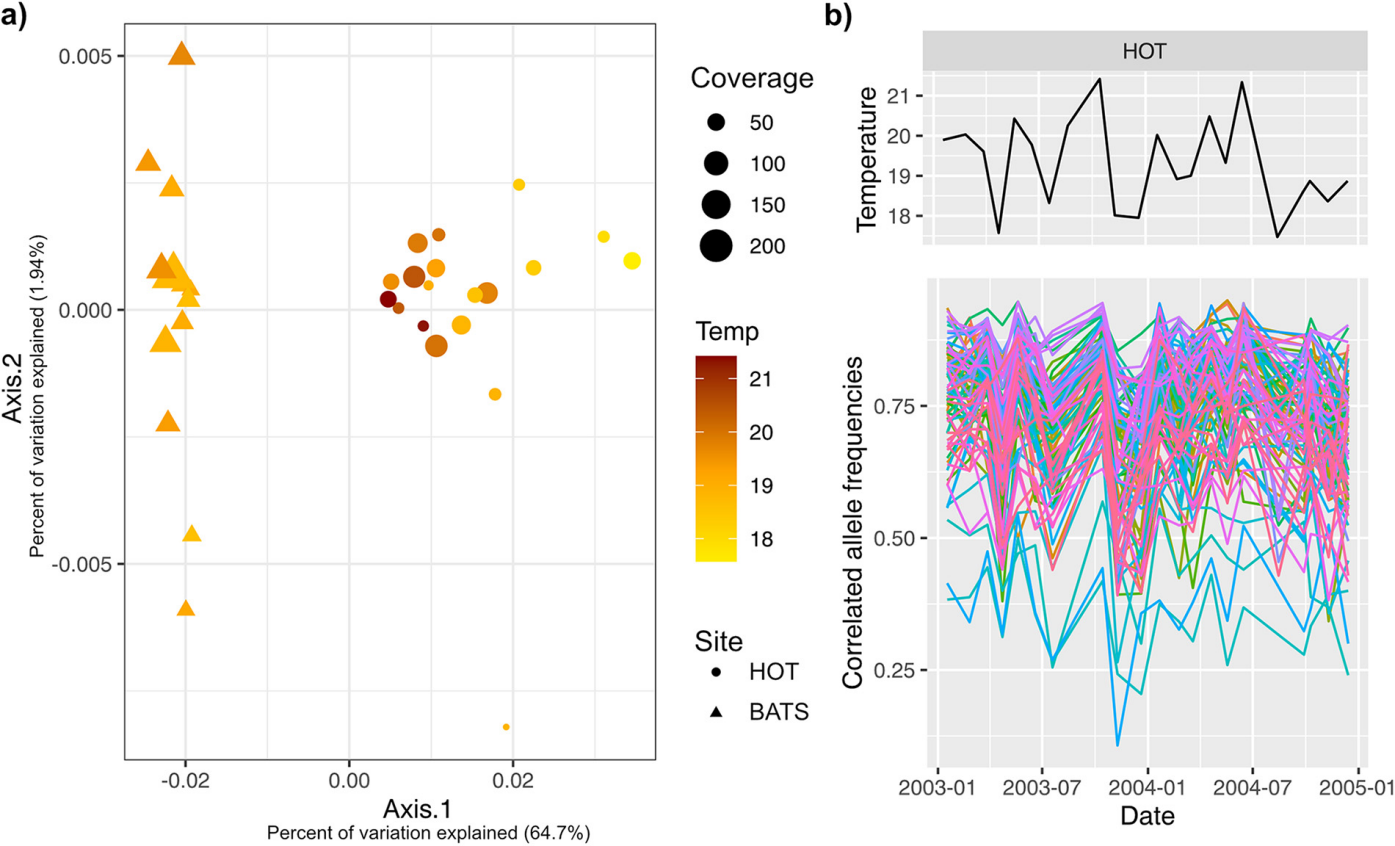
Temperature-correlated variation in allele frequencies of the “*Ca.* N. brevis” population at HOT. (a) Principal-coordinate analysis (PCoA) using pairwise *F_ST_* as a distance metric for BEZ samples at HOT and BATS. “*Ca.* N. brevis” populations in samples collected at different time points are depicted as data points, sized proportionally to the raw coverage. Data points with low coverage (<20×) were excluded from this analysis, and only loci that were detected with a coverage of >20× and within 2 standard deviations from the sample mean across all samples were included in the calculation of pairwise *F_ST_*. The shape and color of the data points correspond to the site and temperature of the corresponding sample, respectively. (b) Frequency fluctuation of temperature-correlated SNVs over the sampling period at HOT. The top panel shows the temperature fluctuation over time, and the bottom panel shows the fluctuation in allele frequencies of all 94 nonsynonymous SNVs (with each color depicting a unique SNV) with frequencies correlated with temperature with statistical significance (adjusted *P* value of <0.05 [Spearman’s correlation]).

We observed greater fluctuations in the temperature and salinity of the BEZ samples from the HOT site than for those from the BATS site (see Fig. S15 at https://doi.org/10.6084/m9.figshare.19358069). In order to quantify the differences in allele frequencies between “*Ca.* N. brevis” populations in different samples, we calculated the *F_ST_* scores between every pair of samples in alleles that were sequenced with a coverage of >20× and within 2 standard deviations from the mean. We observed higher *F_ST_* values between HOT samples than between BATS samples (*t* = 4.7; *P* < 1E−5 [by Welch’s *t* test]) (see Fig. S16 at https://doi.org/10.6084/m9.figshare.19358090). Using the pairwise mean *F_ST_* as a distance metric, we conducted a principal-coordinate analysis (PCoA) and identified clustering of “*Ca.* N. brevis” populations at HOT samples with increasing temperature (Fig. 5a). We identified 94 nonsynonymous SNVs with frequency variation correlated with the temperature fluctuations at HOT with statistical significance (adjusted *P* value of <0.05 [Spearman’s correlation]) (Fig. 5b). The temperature-correlated SNVs were found across 71 genes; the gene harboring the largest number of temperature-correlated SNVs encoded a prohibitin (PHB) domain-containing protein, with 7 out of 16 nonsynonymous SNVs fluctuating in frequencies with correlation with temperature. PHBs are a family of membrane proteins with proposed functions involved in cellular scaffolding and signaling, with lipid- and protein-binding properties (54). Other genes with more than one temperature-correlated SNV included genes with putative regulatory domains, such as a KH (RNA-binding) domain and an HTH (DNA-binding helix-turn-helix) domain, and genes with predicted redox properties, such as vitamin B_12_-dependent ribonucleotide reductase, ATP-dependent desthiobiotin synthetase, and NADH-quinone oxidoreductase subunit L.

## DISCUSSION

### Population-level responses to differentiated selective pressures in the Pacific and Atlantic Oceans.

Characterizing the variation in the genetic diversity of natural microbial populations over space and time is critical for understanding the adaptive strategies and population-level responses to the changing environment. Here, we examined the genetic diversity in populations of “*Ca.* N. brevis,” one of the most abundant archaeal species, with key ecological functions in global carbon and nitrogen cycling. We contextualize our findings with other abundant members of the community and found that some microbial populations exhibit more genetic differentiation between environments. For instance, among the microbial populations surveyed in the BEZ of HOT and BATS (Fig. 1c), two other archaeal populations, Poseidoniia_95_1 and Nitrososphaeria_111_0, showed statistically significant differences (*P* < 0.01 [by a *t* test]) in both nucleotide diversity and *pN*/*pS* ratios between the sites, while no significant differences were detected in the other bacterial populations, Gammaproteobacteria_112_0, SAR324_101_1, and Acidimicrobiia_57_1. Future research should investigate the relationship between the genetic diversity of microbial populations and their ecological roles (55), as well as their genomic features (i.e., natural competence genes [56]), to understand how specific microbial populations evolve across environmental gradients.

### Standing genetic diversity and environmental fluctuations.

How microbial populations develop resilience against environmental fluctuations is an active area of research (57–59) with important environmental as well as biotechnological implications. One of the proposed mechanisms of population-level adaptation to random fluctuations is standing genetic variation (60), in which preexisting genetic variation within the population can allow rapid adaptation to random changes in the environment. In contrast, environmental changes with periodicity, such as seasonality, have also been proposed to decrease genetic diversity by shortening the exclusion times of subpopulations (61). In this study, we observed higher nucleotide diversity in the “*Ca.* N. brevis” population at HOT, where more stochastic environmental fluctuations (as proxied by temperature) were observed. Furthermore, we show population structure clustering with temperature at HOT and identified alleles with frequencies that are highly correlated with temperature. We therefore propose that the higher genetic diversity at HOT is linked to the fluctuating conditions in the below-euphotic zone and that it may provide the standing variation needed for resilience against environmental instability at HOT. Correlations between *in situ* temperatures and sequence heterogeneity, as well as temperature-correlated allele frequency trajectories, have also been observed in marine SAR11 populations (62), suggesting that environmentally mediated fine-scale selection may be a prevalent evolutionary process across cosmopolitan marine microbial populations. Our results can also be compared with those from previous work on the populations of *Prochlorococcus* at HOT and BATS (8), which revealed a similar pattern of higher diversity at HOT and basin-specific genomic islands. Although both “*Ca.* N. brevis” and *Prochlorococcus* populations are more diverse at HOT, the depths at which these populations are found experience very different environmental fluctuation regimes. In shallower depths (SW and DCM), investigated by Kashtan et al. (8), BATS experiences larger seasonal variations, while at the BEZ, HOT experiences more stochastic fluctuations (see Fig. S15 at https://doi.org/10.6084/m9.figshare.19358069). Kashtan et al. (8) attributed the pattern of lower diversity in *Prochlorococcus* at BATS to the shorter exclusion times as a result of stronger seasonality. Our results, in juxtaposition with those of *Prochlorococcus*, suggest that the frequency, periodicity, and range of environmental fluctuation may be critical in determining the evolutionary outcome of genetic diversity (63).

### Environmental control of the pangenome size.

Microbial populations exhibit high levels of gene content variability; however, how the environment controls the evolution of the pangenome remains enigmatic. One hypothesis is that microbial populations that occupy a more diverse set of environments could evolve more open pangenomes in order to rapidly adapt to various selective pressures (46). Here, we show that “*Ca.* N. brevis” at BATS has a larger pangenome than at HOT, with more basin-specific genes. For instance, the “*Ca.* N. brevis” populations at BATS gained different types of high-efficiency phosphorus import systems and lost low-efficiency inorganic phosphorus transport systems, likely in response to the constant phosphorus limitation at BATS (33). We also posit that for “*Ca.* N. brevis,” BATS is likely an environment with more selective constraints than HOT, based on the more frequent detection of signatures of gene-level selective sweeps in “*Ca.* N. brevis” populations at BATS than at HOT. A recent study by Liao et al. (46) investigated the pangenome of the *Listeria* genus across various environments and found that phylogroups that occupy a more diverse range of habitats had more open pangenomes. Our results expand upon this finding by showing that species-level populations occupying environments with differential selective pressures exhibit different sizes of the pangenome and postulating that a larger pangenome is an adaptive response to a more diverse set of selective pressures in an environment. The mechanism of pangenome evolution is an active field of research accelerated by the high-throughput discovery of mobile genetic elements (MGEs). Interestingly, in contrast to *Prochlorococcus*, for which basin-specific genes were organized into genomic islands that are recombined (9, 64), we did not detect a strong organization of basin-specific genes in “*Ca.* N. brevis,” suggesting that there may be a disparity in the mechanisms of gene gain and loss between the two species. However, the cooccurrence of sets of basin-specific genes in “*Ca.* N. brevis” should be further investigated with single-cell sequencing, as shotgun metagenomic sequencing cannot resolve the linkage of gene presence over longer distances in a genome. Importantly, in accordance with a previous survey of various MGEs in *Thaumarchaeota* (65), we did not detect any identifiable MGEs in “*Ca.* N. brevis” MAGs, and as such, “*Ca.* N. brevis” may be employing yet-to-be-characterized mechanisms of genetic material exchange.

### Importance of regulatory genes in adaptation across environmental gradients.

Previous surveys of diversity and ecotyping of microbial populations have utilized functional genes due to the ease and cost-effectiveness of amplicon sequencing of conserved genes of known ecological functions (e.g., see references 29 and 66–68). However, we find that most differentiated alleles between HOT and BATS “*Ca.* N. brevis” populations are enriched in regulatory genes, highlighting the importance of regulatory genes in understanding microbial evolution across diverse environments. Furthermore, many of the alleles with fluctuating frequencies correlating with temperature over time were in genes involved in regulation, and a significant fraction of the basin-specific genes were predicted to have a regulatory function. Our results provide lines of evidence that genetic variation in regulatory genes may be as important to microbial habitat expansion and resilience to temporal environmental fluctuations as modifications in key metabolic enzymes. With the decreasing cost of sequencing, characterization of genetic diversity in these regulatory genes, as well as genes of unknown function, will be critical to our understanding of microbial evolution and ecology.

### Conclusions.

The differences in the “*Ca.* N. brevis” populations of the two oceans exist across multiple dimensions, from population-level diversity to differentiated alleles to basin-specific genes. The key finding in this study is that two strategies of genetic diversification, homologous recombination and gene content variation, may be employed differentially within a population, even at the species level, depending on the environment. “*Ca.* N. brevis” populations at the HOT observatory were characterized by higher genetic diversity, stronger signatures of recombination, and greater fluctuations in population-wide allele frequencies over time. In contrast, “*Ca.* N. brevis” populations at the BATS observatory were characterized by a larger pangenome, with auxiliary genes involved in the transport of phosphorus and other nutrients, redox balance, and transcriptional regulation. Our results further highlight the importance of genetic variation among regulatory genes in “*Ca.* N. brevis” and the potential role that variation plays in ecological success across different locales. This finding prompts future research to expand in scope beyond metabolic genes to include regulatory genes in understanding microbial adaptation and evolution. Furthermore, our results highlight the role of genetic material exchange in thaumarchaeal genome diversification and illustrate our knowledge gap in the lesser-known mechanisms and controls of genetic material exchange in *Thaumarchaeota*. Current and future research on marine *Thaumarchaeota* and other cosmopolitan microbial populations should consider how differential modes of genetic diversification might have shaped genomic heterogeneity and flexibility.

## MATERIALS AND METHODS

### Metagenomic data set and binning of metagenome-assembled genomes.

We used the time series metagenome data set of reads and assembled contigs of HOT (22°45′N, 158°00′W) and BATS (31°50′N, 64°10′W) previously reported by Biller et al. (69). The sampling procedure, library preparation, sequencing, quality filtering, and assembly are described in the original publication (69). We used 64 HOT and 62 BATS metagenomic samples collected approximately monthly between 2003 and 2004 from surface water (SW), the deep chlorophyll maximum (DCM), and the bottom of the euphotic zone (BEZ). Sample metadata can be found in Table S1 in the supplemental material. Assembled contigs with lengths of >1 kbp were used for metagenome-assembled genome (MAG) binning. First, differential coverages of contigs were calculated by all-versus-all mapping of reads against contigs across all samples from each site using Bowtie2 v2.3.2 in sensitive mode (70), which were then filtered at 97% sequence identity. Differential coverages across all 124 samples were used as the inputs for the binning tools CONCOCT (71), maxBin2 (72), ABAWACA (https://github.com/CK7/abawaca), and metaBAT2 (73), and the resulting bins were consolidated using DAS Tool (74). The quality of MAGs was estimated using CheckM v1.1.3 (75).

### Dereplication and annotation of MAGs.

Binned MAGs from both sites were quality filtered at >70% completeness and <10% contamination thresholds and dereplicated using dRep (76) at a 96% average nucleotide identity (ANI) threshold. Dereplicated MAGs were designated representative MAGs (rMAGs). Broad taxonomic classifications of rMAGs were made using GTDB-Tk v1.5.0 classify_wf (77). Of the four thaumarchaeal rMAGs, one was determined to belong to “*Ca.* N. brevis” based on an ANI threshold of >97% using fastANI (78) against the reference genome of “*Ca.* N. brevis” (19). This thaumarchaeal rMAG (rMAG_Nbrevis) and all other MAGs that were clustered with it by dRep were annotated by first predicting the genes using Prodigal v2.6.3 (79) and then aligning the genes using Diamond v2.0.7.145 (80) against the UniRef100 database (81) with an E value cutoff of 1E−5.

### Read mapping, SNV calling, calculation of nucleotide diversity, *pN/pS* ratio (ratio of nonsynonymous to synonymous polymorphisms), linkage disequilibrium, and estimations of the recombination rate, maximal growth rate, and effective population size.

All rMAGs were combined to create a reference genome database against which paired-end reads (150 bp by 2) from each sample were mapped with Bowtie2 in sensitive mode (70). Genome-wide inStrain (42) profiles were created with default settings, except for minimum percent identity filtering at 94%. Relative abundances of rMAGs were determined using the genome-wide average read mapping coverage. Statistical significances of the seasonality (determined by the sample collection month) in the community compositions for each site and depth were determined via PERMANOVA using the adonis function with 999 permutations on the bray distance matrices calculated using the Vegan package in R. Only rMAGs with an average coverage of >5× and breadth (fraction of the rMAG covered by at least one read) of >0.5 were included for relative abundance calculation and further analyses. inStrain profile (42) was used to call SNVs and calculate nucleotide diversity. *pN*/*pS* ratios of genes were calculated for each rMAG in a sample by inStrain profile (42) and were averaged for the calculation of the genome-wide *pN*/*pS* ratio. Linkage disequilibrium (*r*^2^) was calculated for all pairs of SNVs with at least 20 pairs of connecting reads and normalized by rarefying to 20 read pairs using inStrain (42). The recombination rate was inferred using the mcorr package (82) for all populations with >50× coverage, and gamma/mu values above 100 were discarded. The estimated minimal doubling time based on codon usage patterns was calculated using gRodon (83). The lower bounds of the effective population size (*N_e_*) of “*Ca*. N. brevis” were estimated by calculating π_neutral_ in nonconserved third codon positions as previously described by Kashtan et al. (9) In short, the formula *N_e_* = 1.5π_neutral_/(μ(3−4π_neutral_)) (84) was used to estimate *N_e_*, assuming a mutation rate (μ) of 10^−10^ mutations per bp per generation (9, 85). Note that this is a lower bound of *N_e_* because not all third codon positions are neutral, and neutral sites are likely saturated with mutations, thereby underestimating π_neutral_.

### Estimations of pangenome sizes and openness and analyses of basin-specific genes.

Pangenome sizes were compared for HOT and BATS “*Ca.* N. brevis” populations found in the BEZ habitat. We chose to focus on the BEZ samples because of the consistently high abundances of “*Ca.* N. brevis” at this sampling depth over the entire sampling period at both sites. For pangenome construction, we used Roary v3.13.0 (86) with flag -s on quality-filtered (completeness of >70% and contamination of <10%) MAGs binned from BEZ samples and clustered with rMAG_Nbrevis at >96% ANI. All genes in the pangenome were extracted to create a reference database to which all reads across all BEZ samples from HOT and BATS were mapped using Bowtie2 v2.3.2 in sensitive mode (70) and filtering for a maximum of 15 mismatches (>90% sequence identity) per read. Relative abundances of genes were determined by normalizing the coverages of genes by the average coverage of rMAG_Nbrevis in each sample, and genes with a relative abundance of >3 were discarded as genes likely also present in populations other than rMAG_Nbrevis. Variation in relative gene coverage in BEZ samples from HOT versus BATS was assessed via ANOVA (51), and the resulting *P* values were adjusted for multiple-hypothesis tests using the Benjamini-Hochberg procedure (87). Genes with an adjusted *P* value of <0.01 were classified as basin specific if present at coverages of <10% of the average coverage of rMAG_Nbrevis across all time points in either HOT or BATS. Genetic neighborhoods of phosphate and phosphonate import systems were visualized using easyfig v2.2.2 (88) with a BLASTn E value threshold of 1E−5. Phylogenetic trees of the proteins involved in the inorganic phosphate transport (Pit) system were constructed as follows: relevant proteins were clustered based on UniRef100 annotations, and the top 100 BLAST results of the representative sequences against the NCBI nr database were used for alignments. Alignments were made using MUSCLE (89) followed by BMGE v1.12 trimming (90), and the tree was calculated using IQ-TREE v2.1.2 (91) with flags -m MFP -bb 1000 and visualized using iTOL (92). We also calculated the pangenome openness (γ) using a method previously described by Liao et al. (46) for populations for which >15 medium- to high-quality MAGs could be binned across all samples (all depths and sites).

### *F_ST_* calculation and estimation of divergence time between HOT and BATS backbone populations.

The *F_ST_* (fixation index) (measure of differential allele frequencies between populations) value for each “*Ca.* N. brevis” gene was calculated between two metagenomic samples using a method previously described by Crits-Christoph et al. (44). The mean *F_ST_* was calculated for each BEZ sample pair for which rMAG_Nbrevis was detected at >20× coverage, by averaging the *F_ST_* values of genes that were mapped at >20× coverage in all queried samples. The PhoU protein structure was predicted using Phyre2 (http://www.sbg.bio.ic.ac.uk/phyre2) (93) in intensive mode. Pockets were determined using fpocket2 (94) and visualized using PyMOL (Schrödinger, LLC). Pairwise mean *F_ST_* values were used to create a distance matrix between samples, which was then used for principal-coordinate analysis (PCoA) using the pcoa function in R with cailliez correction. The lower bound for the time of divergence between the HOT and BATS backbone populations was estimated by first identifying the SNVs where the consensus alleles of the two populations differ (here, consensus single nucleotide polymorphisms [SNPs]) using inStrain compare (42). We used the method used previously for *Prochlorococcus* by Kashtan et al. (9) to calculate the time of divergence based on the number of consensus SNPs in the third base codons by assuming a constant mutation rate of 10^−10^ mutations per bp per generation and a generation time of 7 days (20).

### Statistical analyses and visualization.

All statistical tests were performed using R v4.0.2 (95) and visualized using ggplot2 (96).

### Data availability.

Metagenomic reads and contigs used in this study can be found under NCBI BioProject accession number PRJNA385855. rMAG_Nbrevis binned in this study is available in Data Set S1 in the supplemental material.

10.1128/msystems.01477-21.5 DATA SET S1 Fasta file of rMAG_Nbrevis. Download Data Set S1, TXT file, 1.1 MB. Copyright © 2022 Hwang and Girguis. 2022 Hwang and Girguis. https://creativecommons.org/licenses/by/4.0/ This content is distributed under the terms of the Creative Commons Attribution 4.0 International license.

## References

[1] Ward BA, Cael BB, Collins S, Young CR. 2021. Selective constraints on global plankton dispersal. Proc Natl Acad Sci USA 118:e2007388118. doi:10.1073/pnas.2007388118. 33649201 PMC7958371

[2] Azam F, Malfatti F. 2007. Microbial structuring of marine ecosystems. Nat Rev Microbiol 5:782–791. doi:10.1038/nrmicro1747. 17853906

[3] Sunagawa S, Coelho LP, Chaffron S, Kultima JR, Labadie K, Salazar G, Djahanschiri B, Zeller G, Mende DR, Alberti A, Cornejo-Castillo FM, Costea PI, Cruaud C, d’Ovidio F, Engelen S, Ferrera I, Gasol JM, Guidi L, Hildebrand F, Kokoszka F, Lepoivre C, Lima-Mendez G, Poulain J, Poulos BT, Royo-Llonch M, Sarmento H, Vieira-Silva S, Dimier C, Picheral M, Searson S, Kandels-Lewis S, Tara Oceans Coordinators, Bowler C, de Vargas C, Gorsky G, Grimsley N, Hingamp P, Iudicone D, Jaillon O, Not F, Ogata H, Pesant S, Speich S, Stemmann L, Sullivan MB, Weissenbach J, Wincker P, Karsenti E, Raes J, Acinas SG, Bork P. 2015. Ocean plankton. Structure and function of the global ocean microbiome. Science 348:1261359. doi:10.1126/science.1261359. 25999513

[4] Biers EJ, Sun S, Howard EC. 2009. Prokaryotic genomes and diversity in surface ocean waters: interrogating the global ocean sampling metagenome. Appl Environ Microbiol 75:2221–2229. doi:10.1128/AEM.02118-08. 19201952 PMC2663191

[5] Biller SJ, Berube PM, Lindell D, Chisholm SW. 2015. Prochlorococcus: the structure and function of collective diversity. Nat Rev Microbiol 13:13–27. doi:10.1038/nrmicro3378. 25435307

[6] Giovannoni SJ. 2017. SAR11 bacteria: the most abundant plankton in the oceans. Annu Rev Mar Sci 9:231–255. doi:10.1146/annurev-marine-010814-015934. 27687974

[7] Banerjee S, Schlaeppi K, van der Heijden MGA. 2018. Keystone taxa as drivers of microbiome structure and functioning. Nat Rev Microbiol 16:567–576. doi:10.1038/s41579-018-0024-1. 29789680

[8] Kashtan N, Roggensack SE, Berta-Thompson JW, Grinberg M, Stepanauskas R, Chisholm SW. 2017. Fundamental differences in diversity and genomic population structure between Atlantic and Pacific Prochlorococcus. ISME J 11:1997–2011. doi:10.1038/ismej.2017.64. 28524867 PMC5563953

[9] Kashtan N, Roggensack SE, Rodrigue S, Thompson JW, Biller SJ, Coe A, Ding H, Marttinen P, Malmstrom RR, Stocker R, Follows MJ, Stepanauskas R, Chisholm SW. 2014. Single-cell genomics reveals hundreds of coexisting subpopulations in wild Prochlorococcus. Science 344:416–420. doi:10.1126/science.1248575. 24763590

[10] Hoarfrost A, Nayfach S, Ladau J, Yooseph S, Arnosti C, Dupont CL, Pollard KS. 2020. Global ecotypes in the ubiquitous marine clade SAR86. ISME J 14:178–188. doi:10.1038/s41396-019-0516-7. 31611653 PMC6908720

[11] Konstantinidis KT, DeLong EF. 2008. Genomic patterns of recombination, clonal divergence and environment in marine microbial populations. ISME J 2:1052–1065. doi:10.1038/ismej.2008.62. 18580971

[12] Rinke C, Chuvochina M, Mussig AJ, Chaumeil P-A, Davín AA, Waite DW, Whitman WB, Parks DH, Hugenholtz P. 2021. A standardized archaeal taxonomy for the Genome Taxonomy Database. Nat Microbiol 6:946–959. doi:10.1038/s41564-021-00918-8. 34155373

[13] Santoro AE, Casciotti KL, Francis CA. 2010. Activity, abundance and diversity of nitrifying archaea and bacteria in the central California Current. Environ Microbiol 12:1989–2006. doi:10.1111/j.1462-2920.2010.02205.x. 20345944

[14] Beman JM, Popp BN, Francis CA. 2008. Molecular and biogeochemical evidence for ammonia oxidation by marine Crenarchaeota in the Gulf of California. ISME J 2:429–441. doi:10.1038/ismej.2007.118. 18200070

[15] Karner MB, DeLong EF, Karl DM. 2001. Archaeal dominance in the mesopelagic zone of the Pacific Ocean. Nature 409:507–510. doi:10.1038/35054051. 11206545

[16] Wuchter C, Abbas B, Coolen MJL, Herfort L, van Bleijswijk J, Timmers P, Strous M, Teira E, Herndl GJ, Middelburg JJ, Schouten S, Sinninghe Damsté JS. 2006. Archaeal nitrification in the ocean. Proc Natl Acad Sci USA 103:12317–12322. doi:10.1073/pnas.0600756103. 16894176 PMC1533803

[17] Shiozaki T, Ijichi M, Isobe K, Hashihama F, Nakamura K-I, Ehama M, Hayashizaki K-I, Takahashi K, Hamasaki K, Furuya K. 2016. Nitrification and its influence on biogeochemical cycles from the equatorial Pacific to the Arctic Ocean. ISME J 10:2184–2197. doi:10.1038/ismej.2016.18. 26918664 PMC4989309

[18] Qin W, Martens-Habbena W, Kobelt JN, Stahl DA. 30 September 2016. Candidatus Nitrosopumilaceae. *In* Trujillo ME, Dedysh S, DeVos P, Hedlund B, Kämpfer P, Rainey FA, Whitman WB (ed), Bergey’s manual of systematics of archaea and bacteria. John Wiley & Sons, Hoboken, NJ.

[19] Santoro AE, Dupont CL, Richter RA, Craig MT, Carini P, McIlvin MR, Yang Y, Orsi WD, Moran DM, Saito MA. 2015. Genomic and proteomic characterization of “Candidatus Nitrosopelagicus brevis”: an ammonia-oxidizing archaeon from the open ocean. Proc Natl Acad Sci USA 112:1173–1178. doi:10.1073/pnas.1416223112. 25587132 PMC4313803

[20] Carini P, Dupont CL, Santoro AE. 2018. Patterns of thaumarchaeal gene expression in culture and diverse marine environments. Environ Microbiol 20:2112–2124. doi:10.1111/1462-2920.14107. 29626379

[21] Qin W, Zheng Y, Zhao F, Wang Y, Urakawa H, Martens-Habbena W, Liu H, Huang X, Zhang X, Nakagawa T, Mende DR, Bollmann A, Wang B, Zhang Y, Amin SA, Nielsen JL, Mori K, Takahashi R, Virginia Armbrust E, Winkler M-KH, DeLong EF, Li M, Lee P-H, Zhou J, Zhang C, Zhang T, Stahl DA, Ingalls AE. 2020. Alternative strategies of nutrient acquisition and energy conservation map to the biogeography of marine ammonia-oxidizing archaea. ISME J 14:2595–2609. doi:10.1038/s41396-020-0710-7. 32636492 PMC7490402

[22] Liu Q, Tolar BB, Ross MJ, Cheek JB, Sweeney CM, Wallsgrove NJ, Popp BN, Hollibaugh JT. 2018. Light and temperature control the seasonal distribution of thaumarchaeota in the South Atlantic bight. ISME J 12:1473–1485. doi:10.1038/s41396-018-0066-4. 29445129 PMC5956005

[23] Hollibaugh JT, Gifford SM, Moran MA, Ross MJ, Sharma S, Tolar BB. 2014. Seasonal variation in the metratranscriptomes of a Thaumarchaeota population from SE USA coastal waters. ISME J 8:685–698. doi:10.1038/ismej.2013.171. 24132081 PMC3930313

[24] Müller O, Wilson B, Paulsen ML, Rumińska A, Armo HR, Bratbak G, Øvreås L. 2018. Spatiotemporal dynamics of ammonia-oxidizing Thaumarchaeota in distinct Arctic water masses. Front Microbiol 9:24. doi:10.3389/fmicb.2018.00024. 29410658 PMC5787140

[25] Hugoni M, Taib N, Debroas D, Domaizon I, Jouan Dufournel I, Bronner G, Salter I, Agogué H, Mary I, Galand PE. 2013. Structure of the rare archaeal biosphere and seasonal dynamics of active ecotypes in surface coastal waters. Proc Natl Acad Sci USA 110:6004–6009. doi:10.1073/pnas.1216863110. 23536290 PMC3625260

[26] Auguet J-C, Casamayor EO. 2013. Partitioning of Thaumarchaeota populations along environmental gradients in high mountain lakes. FEMS Microbiol Ecol 84:154–164. doi:10.1111/1574-6941.12047. 23176712

[27] Tolar BB, Reji L, Smith JM, Blum M, Pennington JT, Chavez FP, Francis CA. 2020. Time series assessment of Thaumarchaeota ecotypes in Monterey Bay reveals the importance of water column position in predicting distribution-environment relationships. Limnol Oceanogr 65:2041–2055. doi:10.1002/lno.11436.

[28] Gubry-Rangin C, Kratsch C, Williams TA, McHardy AC, Embley TM, Prosser JI, Macqueen DJ. 2015. Coupling of diversification and pH adaptation during the evolution of terrestrial Thaumarchaeota. Proc Natl Acad Sci USA 112:9370–9375. doi:10.1073/pnas.1419329112. 26170282 PMC4522744

[29] Sintes E, Bergauer K, De Corte D, Yokokawa T, Herndl GJ. 2013. Archaeal amoA gene diversity points to distinct biogeography of ammonia-oxidizing Crenarchaeota in the ocean. Environ Microbiol 15:1647–1658. doi:10.1111/j.1462-2920.2012.02801.x. 22690844 PMC3712475

[30] Karl DM, Church MJ. 2014. Microbial oceanography and the Hawaii Ocean Time-series programme. Nat Rev Microbiol 12:699–713. doi:10.1038/nrmicro3333. 25157695

[31] Steinberg DK, Carlson CA, Bates NR, Johnson RJ, Michaels AF, Knap AH. 2001. Overview of the US JGOFS Bermuda Atlantic Time-series Study (BATS): a decade-scale look at ocean biology and biogeochemistry. Deep Sea Res 2 Top Stud Oceanogr 48:1405–1447. doi:10.1016/S0967-0645(00)00148-X.

[32] Karl DM, Dore JE, Lukas R, Michaels AF, Bates NR, Knap A. 2001. Building the long-term picture. Oceanography 14:6–17. doi:10.5670/oceanog.2001.02.

[33] Friedman N, Rousso I, Sheves M, Fu X, Bressler S, Druckmann S, Ottolenghi M. 1997. Time-resolved titrations of ASP-85 in bacteriorhodopsin: the multicomponent kinetic mechanism. Biochemistry 36:11369–11380. doi:10.1021/bi970646c. 9298956

[34] Ammerman JW, Hood RR, Case DA, Cotner JB. 2003. Phosphorus deficiency in the Atlantic: an emerging paradigm in oceanography. Eos 84:165–170. doi:10.1029/2003EO180001.

[35] Wu J, Sunda W, Boyle EA, Karl DM. 2000. Phosphate depletion in the western North Atlantic Ocean. Science 289:759–762. doi:10.1126/science.289.5480.759. 10926534

[36] Jickells TD, An ZS, Andersen KK, Baker AR, Bergametti G, Brooks N, Cao JJ, Boyd PW, Duce RA, Hunter KA, Kawahata H, Kubilay N, laRoche J, Liss PS, Mahowald N, Prospero JM, Ridgwell AJ, Tegen I, Torres R. 2005. Global iron connections between desert dust, ocean biogeochemistry, and climate. Science 308:67–71. doi:10.1126/science.1105959. 15802595

[37] Newell SE, Fawcett SE, Ward BB. 2013. Depth distribution of ammonia oxidation rates and ammonia-oxidizer community composition in the Sargasso Sea. Limnol Oceanogr 58:1491–1500. doi:10.4319/lo.2013.58.4.1491.

[38] Dore JE, Karl DM. 1996. Nitrification in the euphotic zone as a source for nitrite, nitrate, and nitrous oxide at Station ALOHA. Limnol Oceanogr 41:1619–1628. doi:10.4319/lo.1996.41.8.1619.

[39] Bowers RM, Kyrpides NC, Stepanauskas R, Harmon-Smith M, Doud D, Reddy TBK, Schulz F, Jarett J, Rivers AR, Eloe-Fadrosh EA, Tringe SG, Ivanova NN, Copeland A, Clum A, Becraft ED, Malmstrom RR, Birren B, Podar M, Bork P, Weinstock GM, Garrity GM, Dodsworth JA, Yooseph S, Sutton G, Glöckner FO, Gilbert JA, Nelson WC, Hallam SJ, Jungbluth SP, Ettema TJG, Tighe S, Konstantinidis KT, Liu W-T, Baker BJ, Rattei T, Eisen JA, Hedlund B, McMahon KD, Fierer N, Knight R, Finn R, Cochrane G, Karsch-Mizrachi I, Tyson GW, Rinke C, Genome Standards Consortium, Lapidus A, Meyer F, Yilmaz P, Parks DH, Eren AM, Schriml L, Banfield JF, Hugenholtz P, Woyke T. 2017. Minimum information about a single amplified genome (MISAG) and a metagenome-assembled genome (MIMAG) of bacteria and archaea. Nat Biotechnol 35:725–731. doi:10.1038/nbt.3893. 28787424 PMC6436528

[40] Partensky F, Hess WR, Vaulot D. 1999. Prochlorococcus, a marine photosynthetic prokaryote of global significance. Microbiol Mol Biol Rev 63:106–127. doi:10.1128/MMBR.63.1.106-127.1999. 10066832 PMC98958

[41] Aylward FO, Santoro AE. 2020. Heterotrophic Thaumarchaea with small genomes are widespread in the dark ocean. mSystems 5:e00415-20. doi:10.1128/mSystems.00415-20. 32546674 PMC7300363

[42] Olm MR, Crits-Christoph A, Bouma-Gregson K, Firek BA, Morowitz MJ, Banfield JF. 2021. inStrain profiles population microdiversity from metagenomic data and sensitively detects shared microbial strains. Nat Biotechnol 39:727–736. doi:10.1038/s41587-020-00797-0. 33462508 PMC9223867

[43] Sjöqvist C, Delgado LF, Alneberg J, Andersson AF. 2021. Ecologically coherent population structure of uncultivated bacterioplankton. ISME J 15:3034–3049. doi:10.1038/s41396-021-00985-z. 33953362 PMC8443644

[44] Crits-Christoph A, Olm MR, Diamond S, Bouma-Gregson K, Banfield JF. 2020. Soil bacterial populations are shaped by recombination and gene-specific selection across a grassland meadow. ISME J 14:1834–1846. doi:10.1038/s41396-020-0655-x. 32327732 PMC7305173

[45] Arnold B, Sohail M, Wadsworth C, Corander J, Hanage WP, Sunyaev S, Grad YH. 2020. Fine-scale haplotype structure reveals strong signatures of positive selection in a recombining bacterial pathogen. Mol Biol Evol 37:417–428. doi:10.1093/molbev/msz225. 31589312 PMC6993868

[46] Liao J, Guo X, Weller DL, Pollak S, Buckley DH, Wiedmann M, Cordero OX. 2021. Nationwide genomic atlas of soil-dwelling Listeria reveals effects of selection and population ecology on pangenome evolution. Nat Microbiol 6:1021–1030. doi:10.1038/s41564-021-00935-7. 34267358

[47] Berube PM, Rasmussen A, Braakman R, Stepanauskas R, Chisholm SW. 2019. Emergence of trait variability through the lens of nitrogen assimilation in Prochlorococcus. Elife 8:e41043. doi:10.7554/eLife.41043. 30706847 PMC6370341

[48] Rosen MJ, Davison M, Bhaya D, Fisher DS. 2015. Microbial diversity. Fine-scale diversity and extensive recombination in a quasisexual bacterial population occupying a broad niche. Science 348:1019–1023. doi:10.1126/science.aaa4456. 26023139

[49] Shapiro BJ, Friedman J, Cordero OX, Preheim SP, Timberlake SC, Szabó G, Polz MF, Alm EJ. 2012. Population genomics of early events in the ecological differentiation of bacteria. Science 336:48–51. doi:10.1126/science.1218198. 22491847 PMC3337212

[50] Vos M, Didelot X. 2009. A comparison of homologous recombination rates in bacteria and archaea. ISME J 3:199–208. doi:10.1038/ismej.2008.93. 18830278

[51] Fisher RA. 1919. XV. The correlation between relatives on the supposition of Mendelian inheritance. Trans R Soc Edinb 52:399–433. doi:10.1017/S0080456800012163.

[52] Kamennaya NA, Geraki K, Scanlan DJ, Zubkov MV. 2020. Accumulation of ambient phosphate into the periplasm of marine bacteria is proton motive force dependent. Nat Commun 11:2642. doi:10.1038/s41467-020-16428-w. 32457313 PMC7250820

[53] Stasi R, Neves HI, Spira B. 2019. Phosphate uptake by the phosphonate transport system PhnCDE. BMC Microbiol 19:79. doi:10.1186/s12866-019-1445-3. 30991951 PMC6469041

[54] Chiba S, Ito K, Akiyama Y. 2006. The Escherichia coli plasma membrane contains two PHB (prohibitin homology) domain protein complexes of opposite orientations. Mol Microbiol 60:448–457. doi:10.1111/j.1365-2958.2006.05104.x. 16573693

[55] Sriswasdi S, Yang C-C, Iwasaki W. 2017. Generalist species drive microbial dispersion and evolution. Nat Commun 8:1162. doi:10.1038/s41467-017-01265-1. 29079803 PMC5660117

[56] Johnston C, Martin B, Fichant G, Polard P, Claverys J-P. 2014. Bacterial transformation: distribution, shared mechanisms and divergent control. Nat Rev Microbiol 12:181–196. doi:10.1038/nrmicro3199. 24509783

[57] Philippot L, Griffiths BS, Langenheder S. 2021. Microbial community resilience across ecosystems and multiple disturbances. Microbiol Mol Biol Rev 85:e00026-20. doi:10.1128/MMBR.00026-20. 33789927 PMC8139522

[58] Shade A, Peter H, Allison SD, Baho DL, Berga M, Bürgmann H, Huber DH, Langenheder S, Lennon JT, Martiny JBH, Matulich KL, Schmidt TM, Handelsman J. 2012. Fundamentals of microbial community resistance and resilience. Front Microbiol 3:417. doi:10.3389/fmicb.2012.00417. 23267351 PMC3525951

[59] Shade A, Read JS, Youngblut ND, Fierer N, Knight R, Kratz TK, Lottig NR, Roden EE, Stanley EH, Stombaugh J, Whitaker RJ, Wu CH, McMahon KD. 2012. Lake microbial communities are resilient after a whole-ecosystem disturbance. ISME J 6:2153–2167. doi:10.1038/ismej.2012.56. 22739495 PMC3504957

[60] Barrett RDH, Schluter D. 2008. Adaptation from standing genetic variation. Trends Ecol Evol 23:38–44. doi:10.1016/j.tree.2007.09.008. 18006185

[61] Barton AD, Dutkiewicz S, Flierl G, Bragg J, Follows MJ. 2010. Patterns of diversity in marine phytoplankton. Science 327:1509–1511. doi:10.1126/science.1184961. 20185684

[62] Delmont TO, Kiefl E, Kilinc O, Esen OC, Uysal I, Rappé MS, Giovannoni S, Eren AM. 2019. Single-amino acid variants reveal evolutionary processes that shape the biogeography of a global SAR11 subclade. Elife 8:e46497. doi:10.7554/eLife.46497. 31478833 PMC6721796

[63] Nguyen J, Lara-Gutiérrez J, Stocker R. 2021. Environmental fluctuations and their effects on microbial communities, populations and individuals. FEMS Microbiol Rev 45:fuaa068. doi:10.1093/femsre/fuaa068. 33338228 PMC8371271

[64] Fernández-Gómez B, Fernàndez-Guerra A, Casamayor EO, González JM, Pedrós-Alió C, Acinas SG. 2012. Patterns and architecture of genomic islands in marine bacteria. BMC Genomics 13:347. doi:10.1186/1471-2164-13-347. 22839777 PMC3478194

[65] Krupovic M, Makarova KS, Wolf YI, Medvedeva S, Prangishvili D, Forterre P, Koonin EV. 2019. Integrated mobile genetic elements in Thaumarchaeota. Environ Microbiol 21:2056–2078. doi:10.1111/1462-2920.14564. 30773816 PMC6563490

[66] Dhillon A, Lever M, Lloyd KG, Albert DB, Sogin ML, Teske A. 2005. Methanogen diversity evidenced by molecular characterization of methyl coenzyme M reductase A (mcrA) genes in hydrothermal sediments of the Guaymas Basin. Appl Environ Microbiol 71:4592–4601. doi:10.1128/AEM.71.8.4592-4601.2005. 16085853 PMC1183284

[67] Wei W, Isobe K, Nishizawa T, Zhu L, Shiratori Y, Ohte N, Koba K, Otsuka S, Senoo K. 2015. Higher diversity and abundance of denitrifying microorganisms in environments than considered previously. ISME J 9:1954–1965. doi:10.1038/ismej.2015.9. 25756678 PMC4542046

[68] Sun Y, Luo H. 2018. Homologous recombination in core genomes facilitates marine bacterial adaptation. Appl Environ Microbiol 84:e02545-17. doi:10.1128/AEM.02545-17. 29572211 PMC5960966

[69] Biller SJ, Berube PM, Dooley K, Williams M, Satinsky BM, Hackl T, Hogle SL, Coe A, Bergauer K, Bouman HA, Browning TJ, De Corte D, Hassler C, Hulston D, Jacquot JE, Maas EW, Reinthaler T, Sintes E, Yokokawa T, Chisholm SW. 2018. Marine microbial metagenomes sampled across space and time. Sci Data 5:180176. doi:10.1038/sdata.2018.176. 30179232 PMC6122167

[70] Langdon WB. 2015. Performance of genetic programming optimised Bowtie2 on genome comparison and analytic testing (GCAT) benchmarks. BioData Min 8:1. doi:10.1186/s13040-014-0034-0. 25621011 PMC4304608

[71] Alneberg J, Bjarnason BS, de Bruijn I, Schirmer M, Quick J, Ijaz UZ, Lahti L, Loman NJ, Andersson AF, Quince C. 2014. Binning metagenomic contigs by coverage and composition. Nat Methods 11:1144–1146. doi:10.1038/nmeth.3103. 25218180

[72] Wu Y-W, Simmons BA, Singer SW. 2016. MaxBin 2.0: an automated binning algorithm to recover genomes from multiple metagenomic datasets. Bioinformatics 32:605–607. doi:10.1093/bioinformatics/btv638. 26515820

[73] Kang DD, Li F, Kirton E, Thomas A, Egan R, An H, Wang Z. 2019. MetaBAT 2: an adaptive binning algorithm for robust and efficient genome reconstruction from metagenome assemblies. PeerJ 7:e7359. doi:10.7717/peerj.7359. 31388474 PMC6662567

[74] Sieber CMK, Probst AJ, Sharrar A, Thomas BC, Hess M, Tringe SG, Banfield JF. 2018. Recovery of genomes from metagenomes via a dereplication, aggregation and scoring strategy. Nat Microbiol 3:836–843. doi:10.1038/s41564-018-0171-1. 29807988 PMC6786971

[75] Parks DH, Imelfort M, Skennerton CT, Hugenholtz P, Tyson GW. 2015. CheckM: assessing the quality of microbial genomes recovered from isolates, single cells, and metagenomes. Genome Res 25:1043–1055. doi:10.1101/gr.186072.114. 25977477 PMC4484387

[76] Olm MR, Brown CT, Brooks B, Banfield JF. 2017. dRep: a tool for fast and accurate genomic comparisons that enables improved genome recovery from metagenomes through de-replication. ISME J 11:2864–2868. doi:10.1038/ismej.2017.126. 28742071 PMC5702732

[77] Chaumeil P-A, Mussig AJ, Hugenholtz P, Parks DH. 2019. GTDB-Tk: a toolkit to classify genomes with the Genome Taxonomy Database. Bioinformatics 36:1925–1927. doi:10.1093/bioinformatics/btz848. PMC7703759 31730192

[78] Jain C, Rodriguez-R LM, Phillippy AM, Konstantinidis KT, Aluru S. 2018. High throughput ANI analysis of 90K prokaryotic genomes reveals clear species boundaries. Nat Commun 9:5114. doi:10.1038/s41467-018-07641-9. 30504855 PMC6269478

[79] Hyatt D, Chen G-L, Locascio PF, Land ML, Larimer FW, Hauser LJ. 2010. Prodigal: prokaryotic gene recognition and translation initiation site identification. BMC Bioinformatics 11:119. doi:10.1186/1471-2105-11-119. 20211023 PMC2848648

[80] Buchfink B, Xie C, Huson DH. 2015. Fast and sensitive protein alignment using DIAMOND. Nat Methods 12:59–60. doi:10.1038/nmeth.3176. 25402007

[81] Suzek BE, Huang H, McGarvey P, Mazumder R, Wu CH. 2007. UniRef: comprehensive and non-redundant UniProt reference clusters. Bioinformatics 23:1282–1288. doi:10.1093/bioinformatics/btm098. 17379688

[82] Lin M, Kussell E. 2019. Inferring bacterial recombination rates from large-scale sequencing datasets. Nat Methods 16:199–204. doi:10.1038/s41592-018-0293-7. 30664775

[83] Weissman JL, Hou S, Fuhrman JA. 2021. Estimating maximal microbial growth rates from cultures, metagenomes, and single cells via codon usage patterns. Proc Natl Acad Sci USA 118:e2016810118. doi:10.1073/pnas.2016810118. 33723043 PMC8000110

[84] Lynch M, Conery JS. 2003. The origins of genome complexity. Science 302:1401–1404. doi:10.1126/science.1089370. 14631042

[85] Lynch M, Ackerman MS, Gout J-F, Long H, Sung W, Thomas WK, Foster PL. 2016. Genetic drift, selection and the evolution of the mutation rate. Nat Rev Genet 17:704–714. doi:10.1038/nrg.2016.104. 27739533

[86] Page AJ, Cummins CA, Hunt M, Wong VK, Reuter S, Holden MTG, Fookes M, Falush D, Keane JA, Parkhill J. 2015. Roary: rapid large-scale prokaryote pan genome analysis. Bioinformatics 31:3691–3693. doi:10.1093/bioinformatics/btv421. 26198102 PMC4817141

[87] Benjamini Y, Hochberg Y. 1995. Controlling the false discovery rate: a practical and powerful approach to multiple testing. J R Stat Soc Series B Stat Methodol 57:289–300.

[88] Sullivan MJ, Petty NK, Beatson SA. 2011. Easyfig: a genome comparison visualizer. Bioinformatics 27:1009–1010. doi:10.1093/bioinformatics/btr039. 21278367 PMC3065679

[89] Edgar RC. 2004. MUSCLE: multiple sequence alignment with high accuracy and high throughput. Nucleic Acids Res 32:1792–1797. doi:10.1093/nar/gkh340. 15034147 PMC390337

[90] Criscuolo A, Gribaldo S. 2010. BMGE (Block Mapping and Gathering with Entropy): a new software for selection of phylogenetic informative regions from multiple sequence alignments. BMC Evol Biol 10:210. doi:10.1186/1471-2148-10-210. 20626897 PMC3017758

[91] Nguyen L-T, Schmidt HA, von Haeseler A, Minh BQ. 2015. IQ-TREE: a fast and effective stochastic algorithm for estimating maximum-likelihood phylogenies. Mol Biol Evol 32:268–274. doi:10.1093/molbev/msu300. 25371430 PMC4271533

[92] Letunic I, Bork P. 2016. Interactive Tree of Life (iTOL) v3: an online tool for the display and annotation of phylogenetic and other trees. Nucleic Acids Res 44:W242–W245. doi:10.1093/nar/gkw290. 27095192 PMC4987883

[93] Kelley LA, Sternberg MJE. 2009. Protein structure prediction on the Web: a case study using the Phyre server. Nat Protoc 4:363–371. doi:10.1038/nprot.2009.2. 19247286

[94] Le Guilloux V, Schmidtke P, Tuffery P. 2009. Fpocket: an open source platform for ligand pocket detection. BMC Bioinformatics 10:168. doi:10.1186/1471-2105-10-168. 19486540 PMC2700099

[95] R Core Team. 2013. R: a language and environment for statistical computing. R Foundation for Statistical Computing, Vienna, Austria.

[96] Wickham H, Chang W. 2012. ggplot2: an implementation of the grammar of graphics (0.9. 3 ed). https://ggplot2.tidyverse.org/.

