# Supplementary material for: Differentiated Evolutionary Strategies of Genetic Diversification in Atlantic and Pacific Thaumarchaeal Populations
Source: mSystems. 2022 Jun 13;7(3):e01477-21. doi: 10.1128/msystems.01477-21 (PMC9239043; doi:10.1128/msystems.01477-21)
Supplement: TEXT S1 [file msystems.01477-21-t0001.docx]

**R1. *Ocean specific genes.***

In addition to ocean-specific phosphate transport genes, we detected 31 other transporter proteins that were differentially present between the two oceans. One lysine transporter, a cation:proton antiporter and six transporters of unspecified substrates were found to be BATS-specific, while an ammonium transporter was HOT-specific. Another large fraction (32 genes) of the differentially present accessory genes included oxidoreductases. We detected eight BATS-specific genes encoding redox enzymes such as NADPH-dependent oxidoreductase, SDR family oxidoreductase, arsenate reductase, fumarate reductase, B12-dependent oxidoreductase and FAD-dependent pyridine nucleotide-disulfide oxidoreductase. Genes involved in regulation, such as transcription factors, DNA/RNA binding proteins, and cell cycle proteins also comprised a large fraction (28 genes) of the differentially present genes. We detected ten BATS-specific regulatory genes, including seven transcription factors, one DNA-methyltransferase and one RNA-binding protein, and one transcription factor specific to HOT.
