# Supplementary material for: Differentiated Evolutionary Strategies of Genetic Diversification in Atlantic and Pacific Thaumarchaeal Populations
Source: mSystems. 2022 Jun 13;7(3):e01477-21. doi: 10.1128/msystems.01477-21 (PMC9239043; doi:10.1128/msystems.01477-21)
Supplement: TEXT S2 [file msystems.01477-21-t0002.docx]

**R2. *Time of divergence between HOT and BATS populations.***

We made pairwise comparisons between consensus sequences of  *Ca.* N. brevis populations at BATS and those at HOT. We detected 4123 (± 929) SNVs in the consensus alleles (consensus SNVs). 1917 (± 459) of consensus SNVs were in the third position of the codons; assuming these sites are undergoing neutral selection, we estimated that the majority subpopulations of *Ca.* N. brevis in the two sites diverged at least hundreds of thousands of years ago. We compared the frequency of consensus SNVs of *Ca.* N. brevis with other populations that were found in both HOT and BATS, and found them to be within the same order of magnitude, indicating relatively recent divergence across these abundant members between the two sites (see **Figure S14** at dx.doi.org/10.6084/m9.figshare.19358066).
